# The Cognitive Consequences of the COVID-19 Pandemic on Members of the General Population in Italy: A Preliminary Study on Executive Inhibition

**DOI:** 10.3390/jcm11010170

**Published:** 2021-12-29

**Authors:** Francesca Favieri, Giuseppe Forte, Francesca Agostini, Jasmine Giovannoli, Enrico Di Pace, Viviana Langher, Renata Tambelli, Mariella Pazzaglia, Anna Maria Giannini, Maria Casagrande

**Affiliations:** 1 Dipartimento di Psicologia, Università di Roma “Sapienza”, Via dei Marsi 78, 00185 Rome, Italy; francesca.favieri@uniroma1.it (F.F.); g.forte@uniroma1.it (G.F.); francesca.agostini@uniroma1.it (F.A.); jasmine.giovannoli@uniroma1.it (J.G.); enrico.dipace@uniroma1.it (E.D.P.); mariella.pazzaglia@uniroma1.it (M.P.); annamaria.giannini@uniroma1.it (A.M.G.); 2 Body and Action Lab, IRCCS Fondazione Santa Lucia, Via Ardeatina 306, 00179 Rome, Italy; 3 Dipartimento di Psicologia Dinamica, Clinica e Salute, Università di Roma “Sapienza”, Via Degli Apuli 1, 00185 Rome, Italy; viviana.langher@uniroma1.it (V.L.); renata.tambelli@uniroma1.it (R.T.)

**Keywords:** COVID-19, pandemic, cognitive functions, inhibition, post-traumatic stress symptoms

## Abstract

The pandemic period which has characterized the last two years has been associated with increasingly worsening psychological conditions, and previous studies have reported severe levels of anxiety, mood disorder, and psychopathological alteration in the general population. In particular, worldwide populations have appeared to present post-traumatic stress symptoms (PTSS). Surprisingly, no studies have evaluated the effect of COVID-related PTSS on cognitive functioning. This study focused on the association between high levels of PTSS related to COVID-19 and alterations in executive functioning by considering executive inhibitions in populations not infected by the virus. Ninety respondents from the Italian population participated in the study. A higher percentage of PTSS was reported. Moreover, respondents with high post-traumatic symptomatology presented deficits in the inhibition of preponderant responses, demonstrating an executive deficit which could be expressed by a difficulty in controlling goal-directed actions. This was underlined by worse performances in elaborating incongruent stimuli in the Stroop task and no-go stimuli in the Go/No-Go task. This report presents preliminary findings underlining the effect of the COVID-19 pandemic on cognitive functions. The results confirmed a persistently higher post-traumatic symptomatology related to the COVID-19 pandemic in the Italian population and highlighted an association with cognitive inhibition impairment.

## 1. Introduction

The effects of the coronavirus (COVID-19) pandemic on mental health were highlighted in the first weeks of the virus outbreak (e.g., [1,2,3,4]). A higher prevalence of PTSD symptomatology [5,6], sleep disturbances [1,7], and psychological distress [3,8] was reported. Generally, negative psychological conditions and worse mental health were found (for a review: [9,10]). These effects should be ascribed to both the direct (i.e., physical and neurological conditions related to inflammatory processes [11]) and indirect (e.g., social distancing measures, fear of the contagion) effects of the pandemic. Specifically, as reported by the systematic review of Vindegaard and Benros (2020), anxiety and depression increased in individuals who contracted the disease as well as in health care professionals with direct contact with the disease. However, as suggested by the authors, the pandemic has also exacerbated pre-existing psychopathological conditions and has generally decreased psychological well-being in the general population due to the aforementioned experiences associated with the spread of COVID-19.

The extensive scientific literature on the COVID-19 pandemic has focused on multiple features related to contagion spread. The epidemiological and clinical features of COVID-19 patients are currently well characterized [12,13], as well as the patients’ mental health conditions [14], the at-risk populations [15], and the general population [16]. However, little attention has been paid to the effects of the pandemic on cognitive functions.

The studies of patients reported cognitive dysfunctions consequent to viral infections [17]. Clinical observations highlighted cognitive alterations (defined as cognitive fog) in patients who contracted COVID-19 [18]. In particular, Almeria et al. [18] reported impaired attention, memory, and executive function in patients affected by COVID-19. Interestingly, these impairments were exacerbated by anxiety and depressive symptoms, suggesting a possible role of some psychological variables. Surprisingly, although a recent review highlighted the detrimental effect of social isolation on cognitive abilities (i.e., executive functions and memory; [19]), only one study [20] focused on the indirect impact of the pandemic on cognitive functions. However, this study assessed only self-reported cognitive functioning; no direct evaluation of performance was made. The risk of contagion appears to be strongly associated with cognitive changes and psychological symptoms (e.g., depression, anxiety, and mood alteration). In Italy, the lockdown and related restrictions have been reported as having an effect on subjective cognitive functioning. Fiorenzato and colleagues identified specific risk factors of worsening cognitive functions related to the COVID-19 lockdown (female gender, young, and having experienced home confinement).

In sum, massive changes to the surrounding context and environment during the COVID-19 lockdown, such as changes in daily routines and the fear of contagion for oneself and others, appear to be related to a compromising of cognitive functions.

Furthermore, exposure to this unprecedented stressful condition has increased the prevalence of mental health disorders, such as depression and anxiety, as well as sleep disorders. It is well-known that these conditions have a substantial impact on cognitive functioning [19].

Considering that the current conditions have lasted longer than expected and that the impact of the situation on cognitive functioning represents a significant public health concern, focusing on these aspects in both the clinical and general population is important for researchers in order to guarantee the well-being of the population. On the one hand, cognitive processes could be affected by the psychological consequences of the COVID-19 pandemic, given the impact of stressful events on the individuals’ cognitive abilities. On the other hand, the uncertainty wrought by the pandemic could affect risk assessments, decision making, and planning processes involving goal-directed executive functioning [21]. Additionally, the pervasiveness and overexposure to pandemic information (e.g., media, social networks, etc.) could affect cognitive functions [22].

Accordingly, the present study sought to explore some cognitive functions—i.e., executive functions—in the general population of Italy during the COVID-19 pandemic. To investigate this topic, we administered a nationwide cross-sectional online survey, which included two cognitive tasks that participants had to perform. Based on previous evidence regarding mental health in the context of the COVID-19 lockdown, we explored COVID-19’s impact on mental health, analyzing the risk of post-traumatic symptomatology among members of the general population who had not been diagnosed with COVID-19. Moreover, we evaluated executive functions with online tasks. According to our previous findings [1,2,7], we expected high COVID-19-related PTSS to be associated with high psychological distress and with worse cognitive performance on tasks meant to assess executive functions.

## 2. Method

### 2.1. Participants

Ninety respondents (women: 70% of the sample; age range: 18–40; mean age: 24.15 ± 2.89) met the inclusion criteria of the study (i.e., no diagnosis of COVID-19, no psychopathological or medical conditions, and no medications) and participated in the study. The main characteristics of the sample are shown in Table 1.

### 2.2. Questionnaires

#### 2.2.1. Demographic Questionnaire and COVID-Related Information

The first section of the survey collected demographic information, including sex (male or female), age, education and occupation, geographical provenience, and medical and psychopathological history. The second section collected information related to participants’ experiences of the pandemic: direct or indirect contact with the virus, close relationships with infected people, close relationships with people in an intensive care unit (ICU), and close relationships with people deceased due to COVID-19.

#### 2.2.2. Post-Traumatic Stress Disorder Related to COVID-19 Questionnaire (COVID-19–PTSD)

The COVID-19-PTSD [5] is a self-report measure designed ad hoc to assess specific symptoms concerning the risk of PTSD related to the COVID-19 pandemic. The questionnaire includes 19 items referring to the previous seven days and requiring a response on a 5-point Likert scale. The COVID-19–PTSD demonstrated excellent internal consistency with regard to the selected items (Cronbach’s α = 0.94). The cut-off of 26 was adopted to split the sample into high and low COVID-19-related PTSD symptomatology (complete information about validation of the questionnaire was reported in [5]).

#### 2.2.3. Symptom Checklist-90 (SCL-90)

SCL-90 [23] assesses psychological distress and symptomatology. It includes 90 items rated on five-point Likert scales, ranging from ‘not at all’ (0) to ‘extremely’ (4). The psychopathological dimensions assessed are Somatization, Obsessive–Compulsive, Interpersonal Sensitivity, Depression, Anxiety, Anger–Hostility, Phobic Anxiety, Paranoid Ideation, and Psychoticism. A Global Severity Index provides a measure of the overall psychological distress. Higher scores in each dimension indicate greater distress and psychopathological symptomatology. The internal consistency of SCL-90 was good for all subscales (α values ranging between 0.70 and 0.96).

### 2.3. Cognitive Tasks

#### 2.3.1. Stroop Task

The Stroop task [24] was adopted to assess executive functions, specifically cognitive inhibition and interference control. The task administered target stimuli consisting of colored words (Font: Arial; Font size: 20; colors: yellow, red, blue, green) that referred semantically to the colors YELLOW, RED, BLUE, and GREEN. Each word could be presented with the ink color related to its semantic meaning (Congruent Condition, e.g., BLUE written in blue ink) or another color (Incongruent Condition, e.g., BLUE written in yellow ink). The task required pressing the key corresponding to the initial word of the ink color in the Italian language (key “R” = red; Key “V” = green; Key “B” = blue; Key “G” = yellow). After a brief presentation of the experiment procedure and examples, a block of 60 randomly presented trials (30 Congruent and 30 Incongruent) was administered. An initial fixation cross (duration: 500 ms) was shown before each trial. The target stimulus remained on the screen for 2000 ms or until the participant’s response. Reaction times (RTs) and the percentage of correct responses were collected for both Congruent and Incongruent trials. The following formula was adopted to compute the Stroop effect: RTs (or % of accuracy) Incongruent Trials—RTs (or % of accuracy) Congruent Trials. A higher Stroop effect in absolute value indicated a greater difficulty in inhibiting inappropriate responses and controlling cognitive interference. Accordingly, more negative values of the Stroop effect indicated inhibitory impairment associated with higher accuracy in congruent conditions or lower accuracy in incongruent ones.

#### 2.3.2. Go/No-Go Task

The Go/No-Go task [25] was adopted to assess motor inhibition, i.e., the ability to control an inadequate motor response. The task included two oval stimuli of different colors (red: No-Go stimuli; green: Go Stimuli) placed in the center of the screen with a black background. The target stimulus (Go) and non-target stimulus (No-Go) were presented randomly on the screen for 2000 ms, followed by a black screen for 500 ms. The participant was required to keep his or her gaze fixed on the center of the screen for the duration of the experiment. The task required participants to press the spacebar as quickly as possible when the green oval appeared in the center of the screen. When the red oval appeared, the participant had to wait for the disappearance of the stimulus. Sixty trials were administered (80% Go trials; 20% No-Go trials), with feedback on correctness. The percentage of inappropriate responses to “No-Go” stimuli (False Alarms) was adopted to assess the inhibition motor component.

### 2.4. Procedure

A web-based cross-sectional survey was implemented using the KoboToolbox Psytoolkit platform and was broadcasted through mainstream social media and message services (such as Facebook, Twitter, Instagram, and Telegram) to collect data. The survey was enabled from September 2020 to November 2020. A brief presentation informed the participants about the aims of the study, and electronic informed consent was required from each participant before starting the investigation. Participants were required to fill out a short demographic questionnaire and respond to questions about their experiences with COVID-19. Then, the SCL-90 was administered. Finally, cognitive tasks were presented via the Psytoolkit server. To guarantee the validity of the cognitive tasks, an accuracy greater than 60 percent represented the criteria for inclusion of data (100% of the data met the inclusion criterion). The total duration of the survey, including cognitive tasks, was about 30 min. To guarantee anonymity, no personal data that could allow the identification of respondents were required. This study was conducted according to the Declaration of Helsinki, and the Ethics Committee of the Department of Dynamic and Clinical Psychology and Health studies (“Sapienza,” the University of Rome, protocol number: Prot. n. 0000515) approved it. Participants could withdraw from the survey at any time without providing any justification, and no data were saved. Only data from completed surveys were considered for the analyses.

### 2.5. Statistical Analysis

A one-way analysis of variance (ANOVA), considering the Group as a between variable, was computed on age. Chi-square comparisons were used to test differences in the distribution of sex, geographical provenience, education and occupation condition, and COVID-19 contact.

A 2 × 2 mixed ANOVA design that considered the Group (high PTSS, low PTSS) as a between-subject variable and the Congruency (Congruent, Incongruent) as a within-subject variable was made on the mean RTs of correct responses and the percentage of accuracy. An ANOVA considering the Group (high PTSD, low PTSD) as a between-subject variable was conducted on the Stroop effect. An ANOVA considering the Group (high PTSS, low PTSS) as a between-subject variable and the percentage of False Alarms as the dependent variable analyzed performance on the Go/No-Go task. Moreover, to evaluate the participants’ psychological conditions, ANOVAs considering the Group (high PTSS, low PTSS) as a between-subject variable and SCL-scores as dependent variables were conducted separately for all SCL scores. Finally, Pearson’s r correlations were adopted to analyze the association between variables. For all the statistical analyses, the level of significance was accepted at *p* < 0.05. The statistical analyses were performed using the Statistica software (version 10.0, Dell, Round Rock, TX, USA).

## 3. Results

There were no significant differences between the groups according to sex, age, geographical provenience, education, or occupation (see Table 1). Considering COVID-19 experiences, the two groups differed in the frequency of direct contact with COVID-19 (X^2^ = 6.96; *p* < 0.03) and relationships with people infected with COVID-19 (Χ^2^ = 4.75; *p* < 0.02). The demographics and COVID-19-related variables of the two groups of participants are shown in Table 1.

### 3.1. Psychological Aspects

Significant differences between groups emerged in all the SCL-90 subscales. In particular, the group with high PTSS reported greater scores than the group with low PTSS in somatization (mean differences: 0.77; F_1,88_ = 28.80; *p* = 0.0001; η^2^ = 0.25), obsessive–compulsivity (mean differences: 0.97; F_1,88_ = 34.62; *p* = 0.0001; η^2^ = 0.28), interpersonal sensitivity (mean differences: 0.81; F_1,88_ = 26.91; *p* = 0.0001; η^2^ = 0.23), depression (mean differences: 1.11; F_1,88_ = 54.66; *p* = 0.0001; η^2^ = 0.38), anxiety (mean differences: 0.98; F_1,88_ = 28.80; *p* = 0.0001; η^2^ = 0.40), anger–hostility (mean differences: 0.73; F_1,88_ = 21.43; *p* = 0.0001; η^2^ = 0.18), phobic anxiety (mean differences: 0.75; F_1,88_ = 29.51; *p* = 0.0001; η^2^ = 0.25), paranoid ideation (mean differences: 0.73; F_1,88_ = 25.09; *p* = 0.0001; η^2^ = 0.22), psychoticism (mean differences: 0.72; F_1,88_ = 33.25; *p* = 0.0001; η^2^ = 0.27), and sleep disturbance (mean differences: 0.78; F_1,88_ = 21.04; *p* = 0.0001; η^2^ = 0.19). Finally, the difference was also confirmed by the SCL-90 global score (mean differences: 0.86; F_1,88_ = 52,60; *p* = 0.0001; η^2^ = 0.37). See Table 2.

### 3.2. Cognitive Functioning

#### 3.2.1. Stroop Task

No significant main effect of the Group (F_1,88_ = 2.27 ; *p* = 0.13 ; η^2^ = 0.03) emerged. A significant main effect of Congruency (F_1,88_ = 27.56; *p* < 0.001; η^2^ = 0.24) revealed that the accuracy was higher in congruent than in incongruent conditions (congruent: 96.99%; incongruent: 93.14%). Finally, the Group x Congruency interaction was significant (F_1,88_ = 10.91; *p* = 0.001; η^2^ = 0.12), underlining that the group with high PTSD reported lower accuracy in incongruent trials (mean differences:−5.30; F_1,88_ = 5.58; *p* = 0.02) compared to the group with low PTSS.

The ANOVA on the Stroop effect revealed a significant difference between the two groups of participants; the Stroop effect was greater in participants with high PTSD compared to those with low PTSS (mean differences:−4.85; F_1,88_ = 10.91; *p* < 0.001; η^2^ = 0.12). Table 3 shows the means (±SD) of the dependent variables of the Stroop task for the two groups of participants.

#### 3.2.2. Go/No-Go Task

Only a marginally significant difference was found in the performance at the Go/No-Go Task (F_1,88_ = 2.91; *p* = 0.09; η^2^ = 0.03). See Table 3.

### 3.3. Correlations

The COVID-19-PTSD score was positively correlated with all the SCL-90 subscales (Pearson’s r ranging from 0.55 and 0.73; *p* < 0.0001). Moreover, the COVID-19-PTSD score was positively correlated with the percentage of False Alarms in the Go/No-Go task (r = 0.26; *p* < 0.01) and negatively with the Stroop effect (r = −0.26; *p* < 0.01).

Negative correlations emerged between the Stroop effect (computed on the accuracy) and the following SCL-90 subscales: somatization (r = −0.31; *p* < 0.003), obsessive–compulsivity (r = −0.22; *p* < 0.03), depression (r = −0.22; *p* < 0.04), anxiety (r = −0.23; *p* < 0.03), anger–hostility (r = −0.22; *p* < 0.03), sleep disturbance (r = −0.20; *p* < 0.05), and the global score (r = −0.24; *p* < 0.02). Positive correlations were found between the percentage of False Alarms in the Go/No-Go task and the following SCL-90 subscales: obsessive–compulsivity (r = 0.26; *p* < 0.01), depression (r = 0.25; *p* < 0.01), anger–hostility (r = 0.30; *p* < 0.004), paranoid ideation (r = 0.33; *p* < 0.002), sleep disturbance (r = 0.25; *p* < 0.01), and the global score (r = 0.24; *p* < 0.02).

## 4. Discussion

This study sought to investigate the cognitive effects of the COVID-19 pandemic and to determine its psychological effects. Our findings confirmed previous evidence showing a worsening of participants’ mental health conditions (e.g., [1,2,3,4,26]). Furthermore, for the first time, the results demonstrated that the COVID-19 pandemic has had a substantial impact on the general health population’s cognitive functioning.

In line with previous studies, 33 percent of the sample reported high PTSD symptomatology as assessed by the COVID-19-PTSD questionnaire [5,27,28]. This high percentage of symptomatology ascribed to the distress reported during the pandemic is very surprising. In fact, the actual data were collected about ten months after COVID-19 first spread and led to the lockdown in Italy. In the first phases (from 18th March to 2nd April) of the virus’ spread, COVID-19-related PTSD symptomatology was present in 29% of the general population of Italy, and it could be ascribed to the sudden, rapid, and unexpected spread of a deadly virus together with the countermeasures (e.g., social isolation, curfew, use of masks, disinfectants, etc.) adopted that suddenly changed peoples’ lifestyles and, for the first time, limited individual freedom. Therefore, post-traumatic symptomatology (characterized by intrusion, avoidance, negative affect, anhedonia, arousal, and maladaptive behaviors [5]) is still present in the general population of Italy, and it increased (4%) a few months after the COVID-19 outbreak. This could be due to the continued alert about the spread of the virus, the fact that vaccines to prevent contagion had not yet been disseminated, and the fact that the government maintained moderate levels of containment by requiring social distancing, restricted entry, and mandatory mask wearing. Furthermore, the data were collected during the second wave of the COVID-19 spread in Italy. This period was characterized by a significant increase in infected people and a high number of deaths. This condition, combined with the persistence of restrictive measures (closures or limitations in the attendance of meeting places, such as schools, universities, cinemas, museums, etc.) adopted to counter the virus’ spread, and linked to the severe economic conditions for many Italians, can explain the high PTSD symptomatology. This study confirms an association between COVID-19-related PTSD symptoms and psychological distress expressed by the psychopathological symptoms assessed by the SCL-90. These findings also prove the association between general psychopathological symptomatology and post-traumatic experience [5] and the higher impact of the COVID-19 pandemic on mental health. The COVID-19 pandemic, unexpectedly and suddenly, has generated fear, has limited individual freedom, and has significantly reduced social interactions. All these aspects appear to be associated with PTSD symptoms [3,4] in the general population, as well as in the absence of concrete consequences of the pandemic, i.e., infection and associated physical symptoms.

Confirmation of the psychological impacts of the pandemic gains additional strength from the effects of the pandemic on cognitive functions. For the first time, our results underlined that the indirect effects of COVID-19, converging in PTSD symptomatology, affected executive functions. As reported regarding previous viral infections (e.g., H1N1, [29]; HIV, [30,31], and according to the results regarding the effect of the SARS-CoV-2 pandemic (for a review: [32]), cognitive impairment was recorded in infected patients, highlighting the role of the inflammatory processes as possible markers of these alterations [33,34,35].

Although cognitive impairment appears to be confirmed in patients affected by the COVID-19 virus, and despite the well-known link between psychological and cognitive functioning, cognitive functions in the general population have not yet been analyzed. Indeed, to our knowledge, only one study [20] has detected subjective cognitive impairment perceived by the general Italian population during the period of restrictions. The adoption of self-reported evaluation represents a limitation of the study. However, the authors demonstrated that the COVID-19 lockdown substantially impacted the subjective perception of the impairment of attention, executive functions, memory, language abilities, and temporal orientation. The results of our study extend this knowledge by providing an objective assessment of the impact of pandemic experiences on cognitive performance, specifically in tasks related to assessing executive functions. Respondents with high post-traumatic symptomatology beyond the threshold indicated by the literature [5] presented deficits in the inhibition of preponderant responses, evidencing an executive deficit which could be expressed by a difficulty in controlling goal-directed actions. On behavioral levels, this was underlined by worse performances in the elaboration of incongruent stimuli in the Stroop task and, even only marginally significant, in the higher number of false alarms in the Go/No-Go task.

Poor executive performance is associated with worse mental health. This result suggests that the high PTSS caused by the COVID-19 pandemic and expressed by an exacerbation of the general psychological symptomatology (e.g., high levels of anxiety, depression, sleep disturbances, etc.) affect cognitive functioning. This association could be explained in light of the highly stressful pandemic situation. Via alterations in central and autonomic responses, distress can negatively affect cognitive functions [36]. Highly emotional and stressful conditions alter the central autonomic network implicated in the optimal inhibitory functioning of the prefrontal cortex, as expressed by poor performance in executive tasks [36,37]. These aspects are confirmed by the prominence of hyperarousal symptoms in PTSD [38], emphasizing how these relationships can also represent a consequence of particularly traumatic events, such as the COVID-19 pandemic.

Despite these interesting findings, the results of this study are preliminary, and some limitations should be highlighted. First, the small sample size may have compromised the statistical power. However, the prevalence of PTSS is in line with previous results from a larger sample during the COVID-19 pandemic, and no previous study has adopted cognitive tasks to measure the impact of the pandemic on executive functions. The young age of the participants may represent another limitation. Because the adoption of the online survey is associated with a selection bias toward younger age groups, the results cannot be generalized. Third, the online survey is subject to data collection bias for both psychological and cognitive dimensions. However, despite the home confinement, and according to previous studies on COVID-19, this methodology represents the best solution. Moreover, a recent review [39] confirmed the validity of the online tools to assess cognitive functioning. The impossibility of controlling the setting makes accuracy a more reliable measure than the more classic recording of reaction times, which are most affected by environmental interference. Fourth, due to the high prevalence of asymptomatic conditions, results may be influenced by a possibly undiagnosed infection not reported by respondents. Fifth, the cross-sectional design did not underline the effective role of the different variables related to the COVID-19 pandemic. Further studies are needed to better understand the relationship between cognitive function and the degree of direct and indirect physical and psychological effects of the virus and the resulting pandemic. For example, more information is needed regarding complex cognitive functions, as well as decision making. A last but important limitation to consider is the definition of PTSS and its association with PTSD diagnoses. We adopted a standardized questionnaire validated to define the risk of COVID-19-PTSD in the general population of Italy. However, a recent overview by Norrholm and colleagues (2021) highlighted the risk of overestimating PTSD diagnoses and the heterogeneity of results in studies that considered the “pandemic event” as criterion A for PTSD. We did not consider a clinical diagnosis of PTSD but rather the presence of PTSS to reduce this risk, but further studies should clarify these disputed aspects.

## 5. Conclusions

This study reports preliminary findings that underline the effect of the COVID-19 pandemic on cognitive functions, considering members of the general, healthy Italian population. The results demonstrate a persistently higher post-traumatic symptomatology related to the COVID-19 pandemic in the Italian population and highlights its association with cognitive inhibition impairment. Moreover, our findings confirm the impact of the pandemic on mental health and cognitive states. Despite some limitations, these results could provide an interesting starting point for further clinical studies. To know that the pandemic had an impact not only at the psychological but also at the cognitive level highlights the importance of an integrated approach to improving the general population’s quality of life. This type of intervention should also consider executive functions, which influence the activities of daily life. Further research is needed to define the short- and long-term consequences of the pandemic on the general population’s cognitive and mental health.

## Figures and Tables

**Table 1:** Demographic and COVID-19 related variables of the two groups of participants.

	Low PTSS (*n* = 60)	High PTSS (*n* = 30)	χ^2^/F	*p*
Age, mean (SD)	24.08 (2.40)	24.30 (3.75)	<1	0.74
Sex, *n* (%)			2.14	0.14
Male	21 (35)	6 (20)		
Female	39 (65)	24 (80)		
Geographical Provenience, *n* (%)			4.04	0.13
North Italy	4 (6.6)	6 (20.0)		
Centre Italy	28 (46.7)	14(46.7)		
South Italy	28 (46.7)	10 (33.3)		
Education, *n* (%)			2.47	0.29
High School degree	23 (38.3)	9 (30)		
College or Master degree	37 (61.7)	21 (70)		
Occupation, *n* (%)			5.86	0.21
Self-employed	2 (3.3)	2 (6.7)		
Employed	7 (11.7)	7 (23.3)		
Unemployed	4 (6.7)	2 (6.7)		
Student	47 (78.3)	19 (63.3)		
Direct contact with COVID-19, *n* (%)			6.96	0.03 *
Yes	7 (11.7)	5 (16.7)		
No	47 (78.3)	16 (53.3)		
Do not know	6 (10)	9 (30)		
Close people infected by COVID-19, *n* (%)			4.75	0.02 *
Yes	34 (56.7)	24 (80)		
No	26 (43.3)	6 (20)		
Close people in ICU for COVID-19, *n* (%)				
Yes	11 (18.3)	11 (36.7)	3.64	0.05
No	49 (81.7)	19 (63.3)		
Close people deceased for COVID-19, *n* (%)			2.13	0.14
Yes	10 (16.7)	9 (30)		
No	50 (83.3)	21 (70)		

SD = standard deviation; PTSS: post-traumatic stress symptoms; ICU: intensive care units. * Level of significance *p* < 0.05.

**Table 2:** Mean and standard deviation in the psychological variables of the two groups of participants.

	Low PTSS (*n* = 60)	High PTSS (*n* = 30)	F	*p*
COVID-19-PTSD	10.87 (7.03)	37.83 (9.05)	241.9	0.0001
SCL-90				
Somatization	0.64 (0.53)	1.41 (0.82)	28.80	0.0001
Obsessive-Compulsivity	0.79 (0.62)	1.77 (0.93)	34.62	0.0001
Interpersonal Sensitivity	0.51 (0.55)	1.32 (0.92)	26.91	0.0001
Depression	0.81 (0.57)	1.93 (0.85)	54.66	0.0001
Anxiety	0.58 (0.44)	1.57 (0.76)	60.06	0.0001
Anger-Hostility	0.52 (0.61)	1.26 (0.88)	21.43	0.0001
Phobic Anxiety	0.51 (0.58)	1.26 (0.68)	29.51	0.0001
Paranoid Ideation	0.44 (0.59)	1.17 (0.87)	25.09	0.0001
Psychoticism	0.29 (0.38)	1.02 (0.81)	33.25	0.0001
Sleep Disturbance	0.82 (0.73)	1.60 (0.81)	21.04	0.0001
Global Index	0.60 (0.43)	1.46 (0.68)	52.60	0.0001

SCL-90: Symptom Checklist-90; PTSS: post-traumatic stress symptoms.

**Table 3:** Mean (± SD) of reaction times and accuracy in the Stroop task and Go/No-Go task for the two groups of participants.

	Low PTSS (*n* = 60)	High PTSS (*n* = 30)	F	*p*
Stroop Task, mean Reaction Tims (ms) (standard deviation)				
Congruent Trials	901.52 (23.06)	953.20 (32.62)	1.67	0.20
Incongruent Trials	974.68 (22.94)	1049.57 (32.44)	3.55	0.06
Stroop effect	73.15 (13.16)	96.37 (18.61)	1.04	0.33
Stroop Task, mean Accuracy (%) (standard deviation)				
Congruent condition	96.7 (6.4)	96.3 (10.7)	<1	0.81
Incongruent condition	95.30 (4.4)	90.0 (16.1)	5.58	0.02 *
Stroop effect	−1.43 (5.64)	−6.29 (8.08)	10.91	0.001 *
Go/No-Go, mean False Alarms (%) (standard deviation)	6.41 (7.73)	9.83 (10.95)	2.91	0.09

* Level of significance *p* < 0.05.
